# Supplementary material for: Resolving the full spectrum of human genome variation using Linked-Reads
Source: Genome Res. 2019 Apr;29(4):635–45. doi: 10.1101/gr.234443.118 (PMC6442396; doi:10.1101/gr.234443.118)
Supplement: Supplemental Material [file supp_gr.234443.118_Supplemental_longranger-2.2.zip › longranger-2.2.tar.bz2!/lr2.2-public/lib/stan/cmdstan/stan/doc/api/html/arg__test_8hpp_source.html]

Stan: src/stan/gm/arguments/arg\_test.hpp Source File


|  |  |
| --- | --- |
|  | Stan  2.5.0  probability, sampling & optimization |


- Main Page
- Related Pages
- Namespaces
- Classes
- Files

- File List
- File Members

All Classes Namespaces Files Functions Variables Typedefs Enumerations Enumerator Friends Macros Pages

- src
- stan
- gm
- arguments

arg\_test.hpp

Go to the documentation of this file.

1 #ifndef STAN\_\_GM\_\_ARGUMENTS\_\_TEST\_\_HPP

2 #define STAN\_\_GM\_\_ARGUMENTS\_\_TEST\_\_HPP

3

4 #include <stan/gm/arguments/list\_argument.hpp>

5

6 #include <stan/gm/arguments/arg\_test\_gradient.hpp>

7

8 namespace stan {

9

10  namespace gm {

11

12  class arg\_test: public list\_argument {

13

14  public:

15

16  arg\_test() {

17

18  \_name = "test";

19  \_description = "Diagnostic test";

20

21  \_values.push\_back(new arg\_test\_gradient());

22

23  \_default\_cursor = 0;

24  \_cursor = \_default\_cursor;

25

26  }

27

28  };

29

30  } // gm

31

32 } // stan

33

34 #endif

35

stan::gm::arg\_test\_gradient

**Definition:** arg\_test\_gradient.hpp:12

stan::gm::list\_argument::\_default\_cursor

int \_default\_cursor

**Definition:** list\_argument.hpp:183

arg\_test\_gradient.hpp

list\_argument.hpp

stan::gm::list\_argument::\_values

std::vector< argument \* > \_values

**Definition:** list\_argument.hpp:185

stan::gm::list\_argument

**Definition:** list\_argument.hpp:12

stan::gm::arg\_test::arg\_test

arg\_test()

**Definition:** arg\_test.hpp:16

stan::gm::arg\_test

**Definition:** arg\_test.hpp:12

stan::gm::argument::\_name

std::string \_name

**Definition:** argument.hpp:76

stan::gm::list\_argument::\_cursor

int \_cursor

**Definition:** list\_argument.hpp:182

stan::gm::argument::\_description

std::string \_description

**Definition:** argument.hpp:77


---

[ Stan Home Page ]

*© 2011–2014,
Stan Development Team.*
